# Gene Expression Alterations and Molecular Analysis of CHEK1 in Solid Tumors

**DOI:** 10.3390/cancers12030662

**Published:** 2020-03-12

**Authors:** Adewale Oluwaseun Fadaka, Olalekan Olanrewaju Bakare, Nicole Remaliah Samantha Sibuyi, Ashwil Klein

**Affiliations:** 1 Bioinformatics research group, Department of Biotechnology, Faculty of Natural Sciences, University of the Western Cape, Private Bag X17, Bellville, Cape Town 7535, South Africa; 2 Department of Science and Technology/Mintek Nanotechnology Innovation Centre, Biolabels Node, Department of Biotechnology, Faculty of Natural Sciences, University of the Western Cape, Private Bag X17, Bellville 7535, South Africa; 3 Plant Omics group, Department of Biotechnology, Faculty of Natural Sciences, University of the Western Cape, Private Bag X17, Bellville, Cape Town 7535, South Africa

**Keywords:** solid tumor, CHEK1, gene expression, molecular docking, cancer, argonaute protein

## Abstract

Alterations in the Checkpoint kinase (CHEK1) gene, its regulation, and the possible clinical outcomes in human solid tumors have not been previously examined. Therefore, the present study was carried out to evaluate the expression of CHEK1 in solid tumors as well as the mechanism by which it can be regulated through non-coding RNAs. The expression of CHEK1 was investigated using Oncomine analysis. cBioPortal, Kaplan–Meier Plotter, and PrognoScan were performed to identify the prognostic roles of this gene in solid tumors. The copy number alteration, mutation, interactive analysis, and visualization of the altered networks were performed by cBioPortal. The molecular binding analysis was carried out by Schrodinger suite, PATCHDOCK, and discovery studio visualizer. The study demonstrated that the CHEK1 gene was differentially expressed in four different cancers, and that reduced CHEK1 mRNA expression is an unfavorable prognostic factor for patients with gastric and colorectal cancer. The molecular docking results showed that the CHEK1 gene can be regulated by microRNAs (miR-195-5p) due to the number of stable hydrogen atoms observed within the distance of 2.0 Å and the favorable amino acids (Ala221, Ile353, Ile365, Ile756, Val797, Val70, Val154, Ile159, Val347, Tyr804, Phe811, Tyr815, and Phe156) identified in the binding pocket of the argonaute protein. Due to the possibility of CHEK1’s involvement in solid tumors, it may potentially be a target for therapeutic intervention in cancer. Further studies into the interaction between CHEK1 and other co-expressed genes may give further insight into other modes of regulation of this gene in cancer patients.

## 1. Introduction

Carcinogenesis is the development of a malignant tumor in healthy tissues resulting from a complex series of events beginning with a single cell that has acquired malignant properties through genetic or epigenetics alterations. Cancers result from a progressive accumulation of genetic alterations (mutations) in many different genes. These mutations include alteration of the copy number, translocation events, base insertion, duplication, deletions, and substitutions [1,2,3]. All cancers, including solid tumors, display recurrent chromosomal abnormalities. However, the exact biological significance relating to gene expression alterations in important genes remains unclear in solid tumors. Advances in the understanding of genetic alterations associated with solid tumors are retarded because of difficulties in the induction of neoplastic cells of many tumors to divide in vitro. Cancer treatments employ DNA-damaging therapies to inhibit tumor cell proliferation and induce cell cycle arrest [4]. Since many solid tumors significantly rely on checkpoint kinase 1 (CHEK1)-mediated cell cycle arrest, especially in the absence of the tumor protein-53 (TP53) gene [5], the CHEK1 gene can be considered as a potential target during the development of novel cancer therapies.

CHEK1, a member of the CHEK family, is a serine/threonine-specific protein kinase known to mediate cell cycle arrest in response to DNA damage [6]. The mechanism of action of this gene involves the activation of ataxia telangiectasia mutated (ATM)/ ataxia telangiectasia and Rad3-related protein (ATR), which triggers the phosphorylation of TP53 and CHEKs. This, in turn, inhibits CDC25 phosphatases, thereby preventing the activation of CDK1/Cyclin B and consequently leading to G2/M arrest and initiation of DNA repair [4,7,8]. It was reported that CHEK1 is altered in 0.80% of all cancers and mutated in 2.62% of malignant solid tumors [9].

More so, the expression of this gene has been implicated in several diseases. In neurodegenerative diseases, CHEK1 abnormalities were associated with dementia and loss of protection in Alzheimer’s disease [10].

CHEK1 is majorly involved in the coordination of DNA repair and therefore is an area of great interest in cancer development and treatment [11]. The CHEK1 gene was previously thought to function as a tumor suppressor because of the regulatory role it plays in DNA damage. However, no evidence of homozygous loss of function mutants for CHEK1 in human cancers was reported [12]. Rather, this gene has been revealed to be overexpressed in several solid tumors. The correlation of CHEK1 expression with tumor grade and disease recurrence was also reported, suggesting its role in tumor development [11,13]. Studies have shown that complete loss of CHEK1 suppresses chemically induced carcinogenesis, and its low expression may result in tumor progression [13].

Although different studies have reported CHEK1 mutations in endometrial, colorectal, and stomach carcinomas with microsatellite instability [14,15,16], the potential role of the CHEK1 gene in the pathogenesis of human cancer is not well defined. CHEK1 has been revealed to be overexpressed in various tumors compared to the adjacent normal tissues [17,18,19,20]. Tumor cells with increased levels of CHEK1 acquire survival advantages due to the ability to resist a higher level of DNA damage. Increased levels of CHEK1 may contribute to chemotherapy resistance [21]. CHEK1 was also regulated at the post-transcriptional level by microRNAs [22,23] which are key regulators of tumor growth and response to cancer treatment [24,25,26]. It is worthwhile to understand the mechanism of microRNA–CHEK1 regulation in solid tumors to provide potential new targets and mechanisms as therapeutic options.

Since the pattern of genetic alterations in human solid tumors is distinct and tends to display multiple clonal structural and numeric chromosome rearrangements, the identification of differentially expressed genes between normal cells and its disease state will be a lead in the identification of genes associated with solid tumors.

Methods, including nucleic acid substitution, serial analysis of gene expression, and differential display, have previously been used to identify cancer-related genes. Lately, the expression of microarray analysis techniques, which allows quantitative and large-scale analysis of gene expression, has been used. Additionally, several web-based databases are being developed to describe new chromosomal abnormalities in the context of tumor histopathology. Therefore, the aim of this study was to evaluate the expression signature, prognostic value, and possible functions of the CHEK1 gene in solid tumors using in silico and molecular docking approaches. Solving cancer problems with genomic data and various in silico approaches is a cost-effective approach in cancer research.

## 2. Results

### 2.1. mRNA Expression Levels of CHEK1 in Cancers (Oncomine)

**Figure 1:**
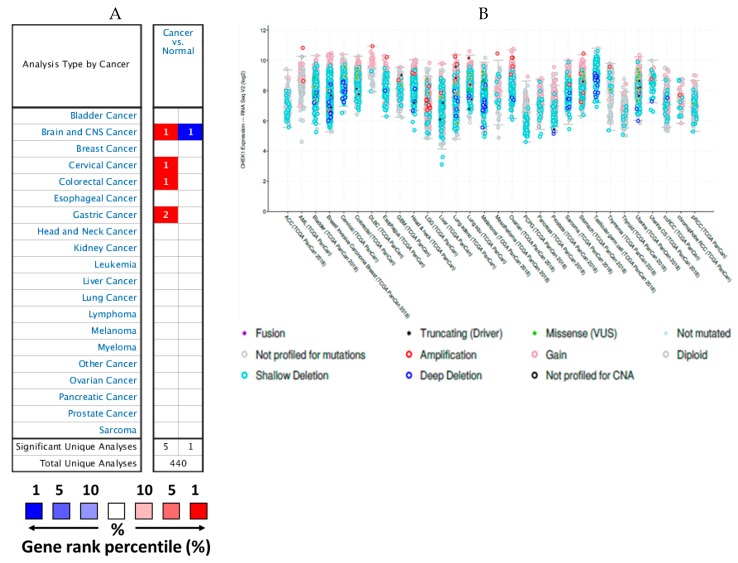
The expression levels of CHEK1 in different types of solid tumors (Oncomine database and cBioPortal). (**A**) This plot indicates the numbers of datasets with statistically significant (p < 0.01) mRNA over-expression (red) or down-expression (blue) of CHEK1 (different types of cancer vs. corresponding normal tissue). The threshold was designed with the following parameters: p-value of 1.0 × 10−8, 2× fold change, and gene ranking of 1%. (**B**) Analysis of CHEK1 mRNA levels in 30 types of human cancers. The median and interquartile ranges are shown in each box.

The transcription levels of CHEK1 were investigated to explore its role in cancer. Relative to normal tissues, the mRNA expression levels of this gene in cancers, especially solid tumors, using the Oncomine database were significantly overexpressed. As shown in Figure 1, it can also be underexpressed, making this gene function as either oncogenic or against oncogenic activities based on the cancer types. Therefore, detailed analyses of CHEK1 were further considered for further analysis.

### 2.2. Expression Levels of CHEK1 in Selected Solid Tumors

**Figure 2:**
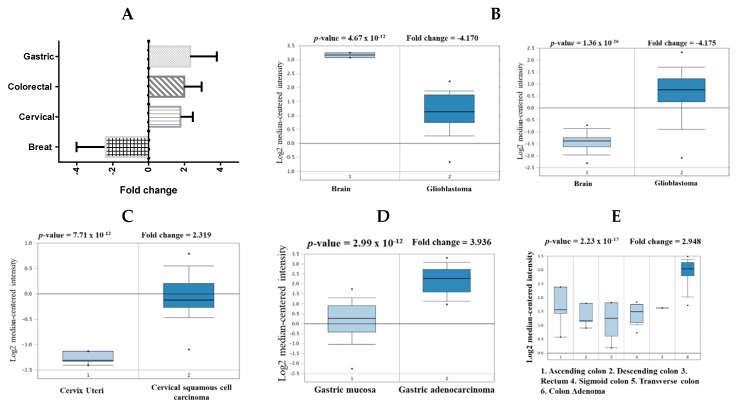
CHEK1 analysis in different cancer types. The box plot comparing specific CHEK1 expression in normal (left plot) and cancer tissue (right plot) was derived from the Oncomine database. The fold change of CHEK1 in various types of cancers was expressed as the forest plot (**A**). The analysis was shown in two sample types, glioblastoma carcinoma relative to normal brain (**B**), in cervical carcinoma relative to normal cervical tissue (**C**), in gastric carcinoma relative to normal gastric tissue (**D**), and in colorectal carcinoma relative to normal colorectum (**E**).

The Oncomine database was used to investigate the transcript expression of CHEK1 in cancers. CHEK1 was used as the query and the result showed that the gene was overexpressed in brain, cervical, colorectal, and gastric cancers and also underexpressed in another brain cancer when compared to the normal counterpart (Table 1, Figure 2).

### 2.3. Survival Analysis

**Figure 3:**
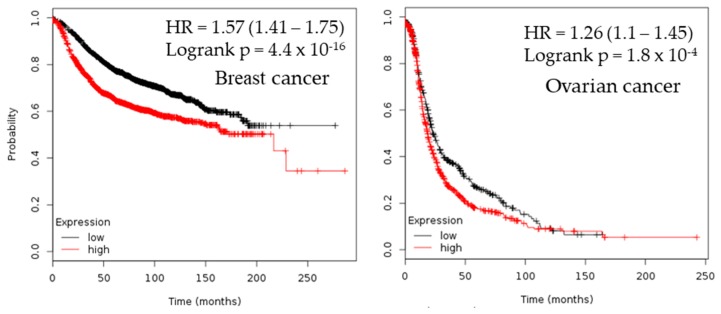

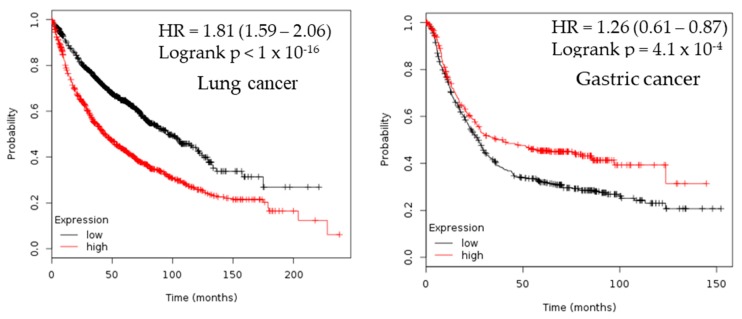
Overall survival analysis of CHEK1. The survival curve comparing the patient with high and low expression in four selected solid tumors was plotted from the Kaplan–Meier plotter database.

Estimation of the prognostic value and overall survival of patients with CHEK1 expression was performed in selected solid tumors. Kaplan–Meier1 Plotter and PrognoScan web-based tools were used to study the expression of the CHECK1 gene with respect to its clinical prognosis. The result shows that downregulation of CHEK1 expression in patients with gastric adenocarcinoma is associated with poor prognosis. Contrastingly, the result obtained in lung adenocarcinoma, ovarian cancer, and breast cancer suggested that high expression of CHEK1 is associated with poor survival rates (Figure 3).

**Figure 4:**
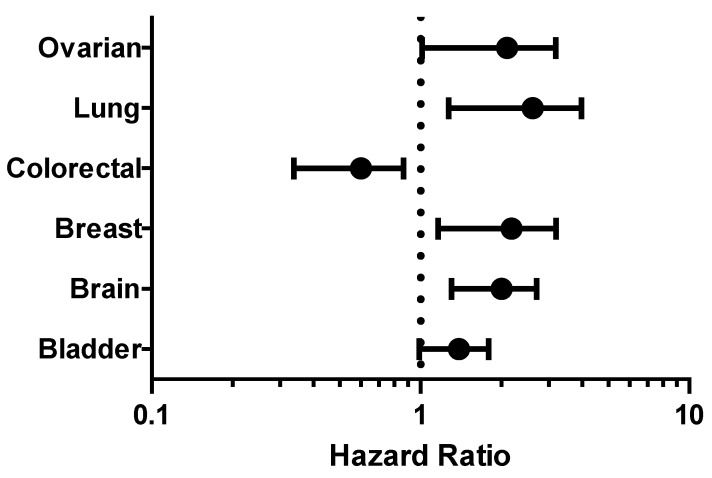
Meta-analysis of CHEK1 genes in different solid tumors. The statistically significant hazard ratio in various types of solid tumors was identified from the PrognoScan database in Table 2 and expressed as the forest plot. The analysis of the survival curve was identified as the threshold of a cox *p*-value < 0.05.

**Figure 5:**
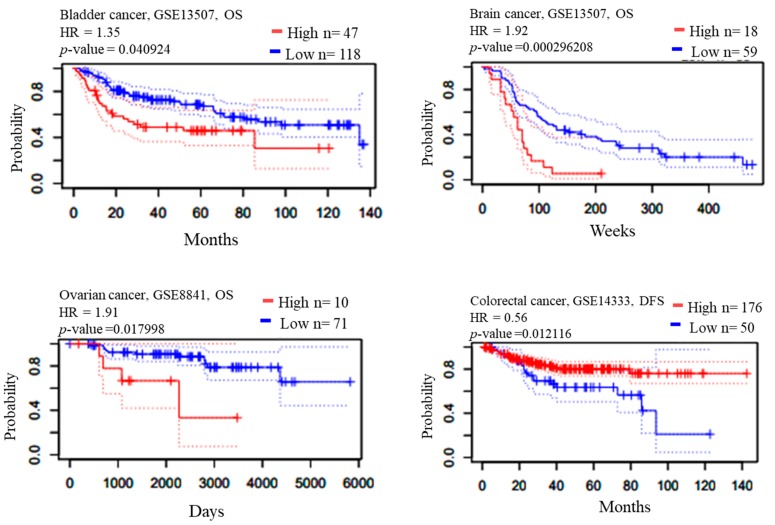

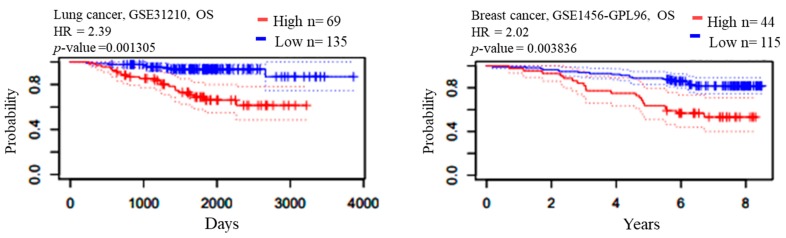
Clinical implication of CHEK1 in bladder, brain, ovarian, colorectal, lung, and breast cancer types. The survival curve comparing the patient with high (red) and low (blue) expression was plotted using the PrognoScan database with the threshold of a cox *p*-value < 0.05. Survival curves illustrate the prognosis of the patients with the CHEK1 gene. The percentage of patients reaching an endpoint was plotted on the vertical axis against time on the horizontal axis using the PrognoScan database to validate the prognostic value of CHEK1 expression in solid tumors (Figure 5).

The prognostic value of CHEK1 expression was reported by the PrognoScan database (Figure 4, Table 2). The poor prognosis in ovarian cancer patients with higher CHEK1 expression (Figure 5) was in line with the data from the Kaplan–Meier plotter analysis (Figure 3). This study therefore employed in silico approaches to demonstrate the oncogenic role of CHEK1 in selected solid tumors

### 2.4. Protein Components of CHEK1

**Figure 6:**
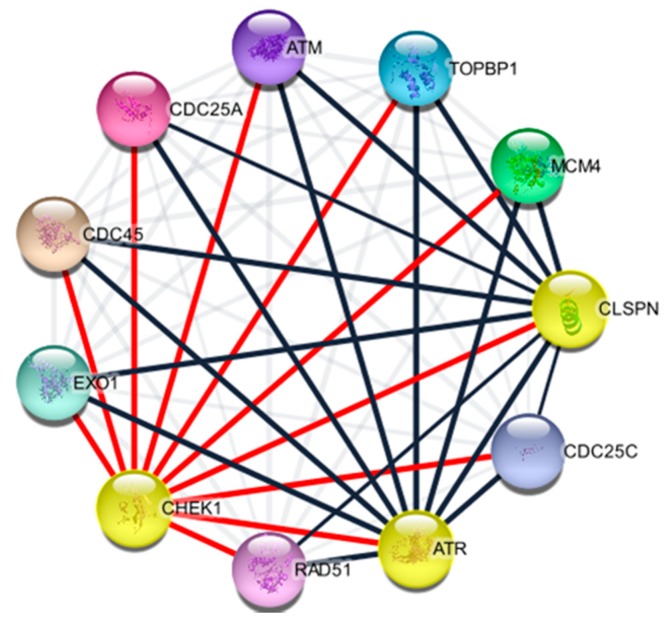
Identification of known and predicted structural proteins essential for CHEK1. The figure consists of 11 nodes and 55 edges, with an average node degree of 6.36 and average local clustering coefficient of 0.855 at a protein-protein (PPI) enrichment *p*-value of 6.94 × 10^−09^. With PPI analysis using STRINGAPP software plugin Cytoscape, all the genes were identified and are relatively important.

Checkpoint kinases are involved in the regulation of DNA replication and cell cycle progression, chromatin restructuring, and apoptosis. Despite these common roles, their biological requirements are different [33]. CHEK1 is mainly involved in mammalian development and viability [34]. Studies revealed that cancer-associated defects of CHEK1 are extremely rare, and so far seem limited to carcinomas of the colon, stomach, and endometrium [14,15,16]. Cytoscape stringAPP plugin was used to determine the protein–protein interaction of CHEK1 and its protein partner. This prediction was based on the curated database, experiment, text-mining, and co-expression/co-occurrence. The 10 predicted proteins of CHEK1 (with the corresponding gene names) including the predicted genes are ATM, cell division cycle 25A and C (CDC25A and C), exonuclease 1 (EXO1), claspin (CLSPN), RAD51recombinase (RAD51), ATR, topoisomerase (DNA) II binding protein 1 (TOPBP1), minichromosome maintenance complex component 4 (MCM4), and cell division cycle 45 (CDC45). These genes were further considered for downstream analysis of CHEK1 (Figure 6).

### 2.5. Genetic Alterations of CHEK1

**Figure 7:**
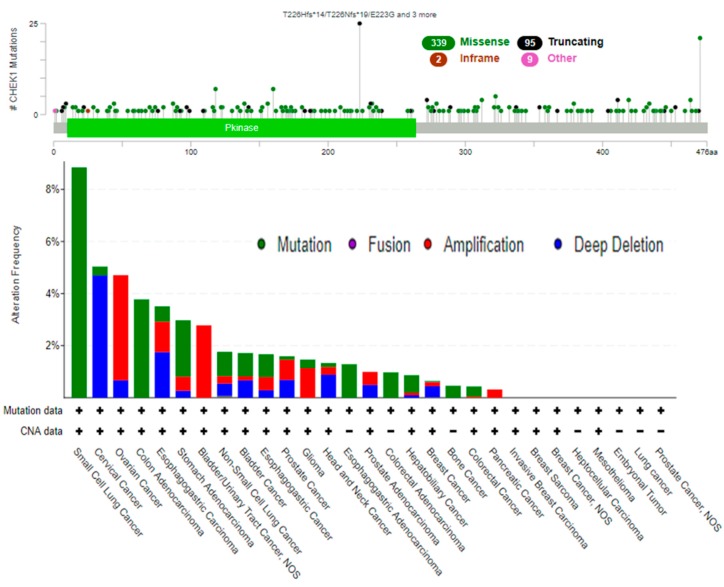
Mutation hotspot of CHEK1 across cancer studies. A total of 445 mutation sites were detected and located between amino acids 0 and 300. CHEK3 mutation mainly occurred in lung cancer and existed in a hotspot in the Pkinase domain.

The genetic alterations of CHEK1 were analyzed in all the different cancers available in the cBioPortal database and the results were compared with those of other genes of interest in Figure 6. The database was queried for CHEK1 gene mutations in 82,899 samples from 275 studies that covered the entire set of available cancers (Figure 7).

**Figure 8:**
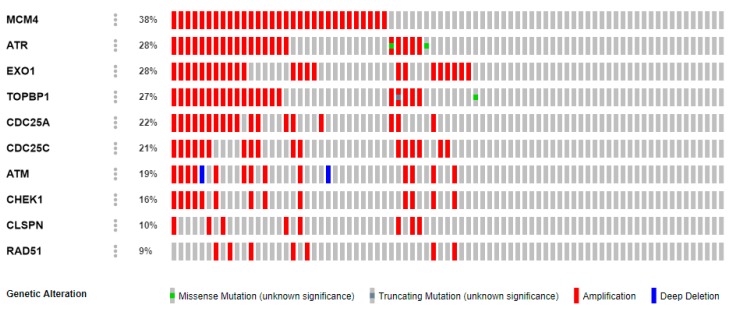
Prostate cancer types frequently amplify CHEK1. Genetic landscape (Oncoplot) of 10 genes in a prostate cancer (Multi-Institute, Nat Med 2016) sample. The figure is a schematic representation (Oncoplot) of the most commonly mutated genes. Each column represents a patient. Colors depict the type of mutations for each gene. Complete samples (114 patients/samples). Queried genes are altered in 51 (45%) of the queried patients/samples.

Additionally, all 10 genes were used as a query to investigate the mutations and copy number alterations (CNAs) in 12 solid tumor studies. The results are presented in Table 3 and Figure 8. The highest frequency of alteration is implicated in prostate cancer.

### 2.6. Co-Expression Profile of CHEK1 in Colorectal Cancer

**Figure 9:**
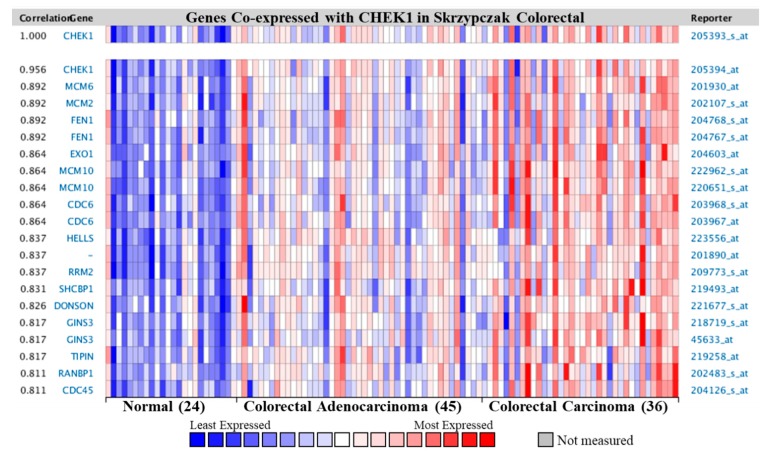
Co-expression profile of CHEK1 in colorectal cancer. CHEK1 is co-expressed with the indicated genes across a panel of 36 colorectal carcinomas and 45 colorectal adenocarcinomas. Bar length represents the significance and negative logarithm of the enrichment *p*-value.

Additionally, the co-expression profiles for CHEK1 with 20 genes across 36 colorectal carcinomas, 45 colorectal adenocarcinomas, and 24 normal colorectum tissues were investigated (Figure 9). Interestingly, CHEK1 was co-expressed with minichromosome maintenance complex component (MCM family) and flap eendonuclease 1 (FEN1) expressed in colorectal cancer. The MCM family (MCMs) has been reported to be associated with numerous cancer types [35,36,37], such as the promotion of metastasis of liver cancer through the Mitogen-activated protein kinase kinase(MEK)/ extracellular signal-regulated kinases (ERK) pathway (MCM6) [38], as a prognostic factor in patients with lung squamous cell carcinoma [39]. Many studies indicate the central roles of MCMs in genome stability, including the regulation of transcription, chromatin remodeling, and checkpoint responses [40,41,42]. Overexpression of MCMs is clinically correlated with cervical carcinogenesis [43]. On the other hand, FEN1 is a multifunctional structure-specific nuclease that has a critical role in maintaining human genome stability. Overexpression of this gene correlates with enhanced proliferation and poor prognosis of lung cancer [44]. The exact underlying mechanism through which CHEK1 modulates cancer progression needs to be further investigated.

### 2.7. Structural Preparation of miR-195-5p-CHEK1 and Human Argonaute Protein Preparation

**Figure 10:**
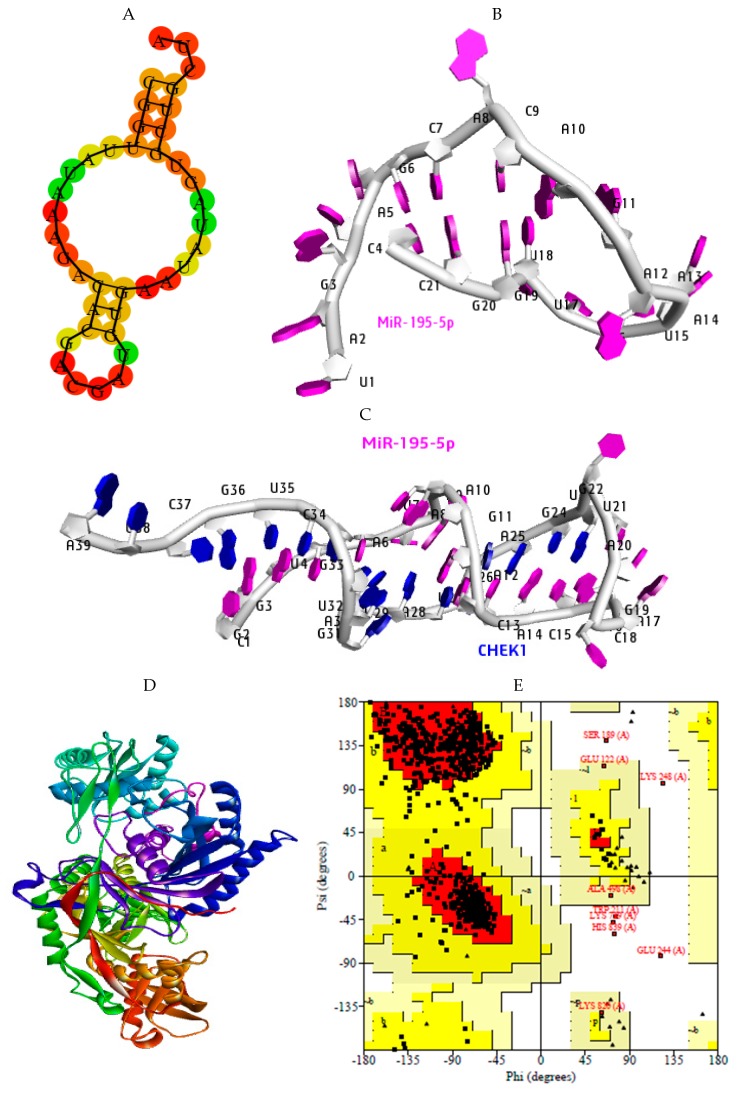
Structural model for molecular docking. (**A**) Secondary structure of MiR-195-5p, 3D structures of (**B**) MiR-195-5p, and (**C**) MiR-195-5p-CHEK1. (**D**) Prepared human argonaute protein (Ago) and (**E**) Ramachandran plot (PDBSum, PROCHECK). The quality of the prepared Ago was estimated by the PDBSum server. The residues in most favored regions (A, B, L), the residues in additional allowed regions (a, b, l, p), and residues in generously allowed regions (~a, ~b ~l, ~p). The structural details of Ago (ID: 4F3T: A) consist of 7 sheets, 9 gamma turns, 13 beta hairpins, 7 beta bulges, 37 strands, and 68 beta turns.

The structural model of miR-195-5p and miR-195-5p-CHEK1 (miRNA-mRNA) duplex (ligands) was built from the miRTarBase, RNAfold, and RNA COMPOSER databases accordingly (Figure 10A–C). The 3D structure of human argonaute protein (receptor) was retrieved from the protein data bank (PDB) and prepared using the Schrodinger suite (Figure 10D). PROCHECK through the Ramachandra plot was used to validate the quality before binding (Figure 10E). Furthermore, the molecular docking of microRNA–argonaute and microRNA–mRNA-bound argonaute was carried out using PATCHDOCK at an RMSD of 1.0 to investigate the mechanism of CHEK1 regulation in solid tumors. The result generated was analyzed in MAESTRO, a Schrodinger software, and visualized using the discovery studio visualizer (DSV).

### 2.8. Molecular Docking Analysis

**Figure 11:**
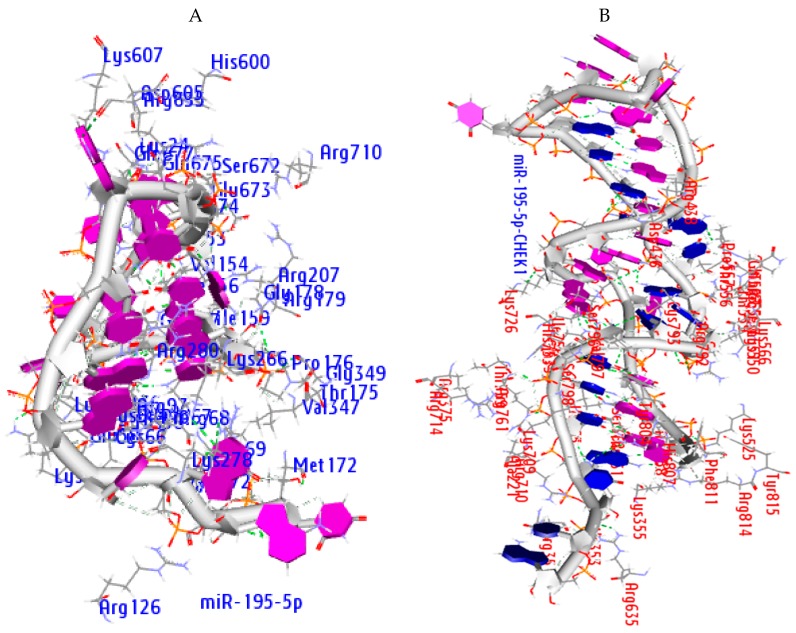
The amino acid residues of human argonaute protein participating in the binding analysis with each mRNA and microRNAs within a distance of 3.5 Å. (**A**) Amino acids participating in miR-195-5-AGO complex, (**B**) amino acids participating in miR-195-5p-CHEK1 duplex and AGO.

A web-based docking tool (PATCHDOCK) was used to study the mode of action of CHEK1 regulation by microRNAs in solid tumors. The PATCHDOCK method is based on the shape complementarity theory [45]. Criteria, such as strong hydrophobic amino acids together with aromatic amino acids, are important to binding interactions in terms of the stability between protein receptors and their ligands. The outputs were ranked according to their geometric shape complementarity score (Table 4). The strong interactions observed were based on the microRNA minimum folding energy of -14kcal/mol (miRTarBase), scores, and the specific amino acid residues involved in binding. As evident, the presence of strong hydrophobic and aromatic amino acid side chains (3.5 Å) (Figure 11; Table 5) and H-bonds (2.0 Å) is proof that CHEK1 regulation through the human argonaute protein could be driven by miR-195-5p (Table 6).

### 2.9. Hydrogen Bond Interaction

Hydrogen bonds (H-bond) play some roles in protein–ligand stability. These roles include orientation of the ligand in the receptor cavity (the pose), ligand recognition by protein, and binding affinity. As one of the contributors to the docking score, the hydrogen bond interactions in the binding pocket of the human argonaute protein when complexed with both microRNA and microRNA-mRNA duplex were evaluated.

**Figure 12:**
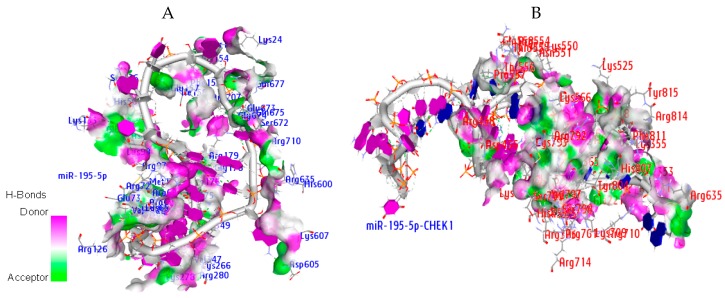
Hydrogen bond interaction between (**A**) the residual amino acids of human argonaute protein and miR-195-5p, and (**B**) the residual amino acids of human argonaute protein and miR-195-5p-CHEK1 duplex (3.5 Å distance).

This bond predicts the specificity of small molecule binding and its important contribution is explicitly incorporated into the molecular interaction to strengthen the binding of ligands to their receptors in an energetically favorable manner. The (H bond) interactions were detected between the amino acid residues of AGO and atoms of miR-195-5p and miR-195-5p-CHEK1 (Figure 12A and B). with a distance of 2.5 Å. Table 6 shows the residues of the amino acids involved in the H-bond between the AGO binding pocket and miR-195-5p and miRNA-mRNA within the distance of 2.0 Å. The results showed that H-bonds increase with atomic interactions. This result may, therefore, support the mode of action of miR-195-5p regulation in the expression of CHEK1 through the RNA-induced silencing complex.

## 3. Discussion

CHEKs belong to the mammalian Ser/Thr kinase protein family, which has two members: CHEK1 and CHEK2. CHEK1 activation must be timely regulated to ensure its proper functioning. The essential mechanism controlling CHEK1 regulation is the phosphorylation of Ser317 and Ser345 at the C-terminal domain, which leads to catalytic activation [46] catalyzed by ATR and ATM kinases [47,48,49]. This study extensively investigated the role of CHEK1 in solid tumors as well as the mechanism by which it can be regulated through molecular docking with human argonaute protein assisted by a known microRNA found to be crucial in cancer (miR-195-5p).

To understand the role of checkpoints in solid tumors, the transcription levels of CHEK1 in normal and cancer tissues were evaluated in the Oncomine database. The various underlying threshold parameters were set as follows: *p*-value of 1.0 × 10^−8^, 2× fold change, and gene ranking of 1%. CHEK1 was found to be overexpressed in some cancer tissues when compared to normal tissues.

The cancer genome atlas contains a large collection of RNA sequencing data and is a useful tool to explore the molecular basis of cancer [50]. By accessing TCGA data via cBioPortal, the mRNA levels of CHEK1 in various solid tumors were found to be differentially expressed in many cancer types (Figure 1b). These were the basis for further downstream analyses of CHEK1.

CHEK1 gene expression was compared from a total of 440 different analyses, and the data was collected from various studies deposited in the Oncomine database. Based on the defined parameters, CHEK1 expression was statistically significant in six studies, and it was reported to be upregulated in brain, central nervous system (CNS), cervical, CRC, and gastric cancers in five studies; in contrast, one of the studies reported reduced expression of CHEK1 in brain and CNS cancers. The majority of previous studies reported that CHEK1 expression is upregulated in various cancers [19,47,51,52], which is in agreement with this study. Various mechanisms involved in the regulation of CHEK1 expression and activation were previously described [53,54,55]. It can also be regulated at the post-transcriptional level by microRNAs [23,56], which are key regulators of tumor growth and response to treatment [57]. The expression of CHEK1 was further validated, and demonstrated that the CHEK1 gene may possess either oncogenic or anti-oncogenic characteristics, depending on the type of cancer.

StringAPP plugin Cytoscape prediction indicated that CHEK1 interacts with 10 other proteins, namely: Ataxia telangiectasia mutated (ATM), cell division cycle 25A and C (CDC25A and C), exonuclease 1 (EXO1), claspin (CLSPN), RAD51 recombinase (RAD51), ataxia telangiectasia and Rad3-related protein (ATR), topoisomerase II binding protein 1 (TOPBP1), minichromosome maintenance complex component 4 (MCM4), and cell division cycle 45 (CDC45). These genes (Figure 6) were predicted based on co-expression/occurrence, experimental, text-mining, or curated databases. Further research is recommended to give insight into the relationship of each gene with respect to CHEK1 in solid tumors.

The CHEK1 gene was altered in 1018 of the queried samples, with a somatic mutation frequency of 1%. As shown in Figure 7, 445 mutations, including 141 duplications, were identified in patients with multiple samples. The alteration sites were detected within a range of 0 to 300. Out of the alterations, 339 missense mutations, 2 in-frame mutations, and 95 truncating mutations were detected. The CHEK1 mutations primarily occurred in lung cancer, spanning the Pkinase domain, with the hotspots in T226Nfs*19, T226Hfs*14, and E223G. The hotspot mutations are likely to support tumor development.

The prognosis analysis result showed that reduced CHEK1 mRNA expression is an unfavorable prognostic factor for patients with gastric and colorectal cancer. Furthermore, the Kaplan–Meier result was in agreement with the PrognoScan result of lung, ovarian, and breast cancer. The probability of a reduced survival rate is greater for patients with high CHEK1 gene expression in bladder, brain, lung, ovary, and breast cancers as compared with patients with low CHEK1 expression. This, therefore, indicated that comprehensive utilization of bioinformatics analyses is a great approach to study the prognosis of genes in solid tumors and may be useful to assess the prognosis for several cancer types.

The transition from a normal cell to a cancer cell is caused by four main events, namely proteomic, transcriptomic, epigenetic, and somatic-acquired genetic alterations [58]. The oncogenic or suppressive roles of these alterations occur in specific genomic regions [59]. Using cBioPortal, a significant copy number alteration in the chosen CHEK1 signature was identified. The analysis queried 10 genes against the prostate cancer dataset in cBioPortal as shown in Figure 8, and the frequency of alteration of these genes was 45%. MCM2 had the highest frequency of alteration. Amplification was the main alteration in all 10 genes, including CHEK1, with 16% of the gene altered through amplification. Other minor alterations observed include missense mutation, truncating mutation, and deep deletion. Gene amplification is commonly observed in some solid tumors and has been implicated in tumor formation [60,61]. Normal cells use the mechanism of gene amplification to overexpress specific genes for survival under unfavorable conditions, such as during exposure to cytotoxic drugs. This mechanism is a typical genetic alteration in cancer, and historically, many oncogenes have been identified in the amplified regions [62]. In light of this, novel cancer-related genes may still be identified in the amplified regions. Previous studies have shown that CHEK1 is involved in various tumors by influencing the target genes or signal pathway, which is consistent with this study [12,63,64,65,66].

Co-expression analysis revealed that CHEK1 was co-expressed with MCM6 in CRC (Figure 9), as well as with EXO1 (Figure 6). Previous studies indicated that MCM2 is upregulated in CRC [67], and the possible association of EXO1 in CRC [68]. The MCMs could potentially be used as a diagnostic marker. This study, therefore, suggests the significance of the co-expressed genes alongside CHEK1 in CRC.

The quality of the prepared human argonaute protein was confirmed by evaluating the stereochemical quality of the protein structure with PDBSum (PROCHECK). This suite uses the Ramachandran plot for structural verification [69]. The output of this analysis proved that the human argonaute protein was fully prepared, with 89.6% of its amino acid residues located in the right region (Figure 10A and B). The model protein was used in the molecular docking analysis to study the mode of regulation of CHEK1 in solid tumors using microRNA. The important amino acids as well as H-bonds within a specific distance using the human argonaute protein (AGO) and miR-195-5p were investigated. Argonautes use small RNA guides to recognize target genes. Human argonaute protein (4F3T) was used for the binding study [70]. MicroRNAs are thought to be crucial in various physiological and pathological processes, especially in cancer [71,72,73,74,75]. MiR-195-59 has been previously studied in solid tumors [76,77,78]. In prostate cancer, miR-195 inhibits tumor progression [79,80]. Other solid tumors in which this microRNA have been investigated in include CRC [81,82], lung cancer [76,83,84,85], gastric cancer [86], cervical cancer [87], breast cancer [88], etc. The common bonds found between proteins and their corresponding molecules include hydrophobic amino acids and H-bonds.

The docking analysis indicated that the non-covalent interactions between the interacting amino acid atoms of AGO and specific atoms of miR-195-5p and/or CHEK1 include hydrophobic interactions. The H-bonds present in the active site of AGO showed that miR-195-5p is essential to CHEK1 regulation by providing stability and steadiness.

The strong binding affinity of these results was observed through their scores and the amino acid residues involved in the interaction between CHEK1, miR-195-5p, and AGO. The amino acids (Ala221, Ile353, Ile365, Ile756, Val797, Val70, Val154, Ile159, Val347, Tyr804, Phe811, Tyr815, and Phe156) and H-bonds within a distance of 2.0 Å supported that CHEK1 regulation through the human argonaute protein (4F3T) was driven by miR-195–5p.

## 4. Materials and Methods

### 4.1. CHEK1 Expression Analysis (Oncomine Database)

Analysis of CHEK1 transcription levels in various solid tumors was performed by the web-based cancer microarray database Oncomine at https://www.oncomine.org/resource/login.html. mRNA expression of clinical tumor and normal samples was compared using a Students’ *t*-test to generate a *p*-value. The threshold search criteria used were a *p*-value < 1 × 10^−8^, a fold change > 2, and a gene rank in the top 1% to obtain the most significant STAT3 probes. A heat map was used to define the co-expression profiles of the CHEK1 gene in different types of cancers. The co-expression profiles of CHEK1 in different cancer types were also extracted from Oncomine and are illustrated as a heat map.

### 4.2. Identification of CHEK1 Associated Proteins

Search Tool for the Retrieval of Interacting Genes (STRING), accessed at http://string-db.org/, was used to predict interactive proteins using CHEK1 as a query. STRING (v 11.0) is a unique tool that provides a comprehensive view of all the known and predicted interactions and associations among proteins [89]. The output is in the form of a network showing the relationships between genes in the list, where nodes symbolize genes and links represent networks at a confidence level of 0.90. This network was visualized with stringAPP, a plugin for Cytoscape v3.7.2 (http://www.cytoscape.org/) [90,91].

### 4.3. Prognostic Analysis of CHEK1

PrognoScan, accessed at http://dna00.bio.kyutech.ac.jp/PrognoScan/, provides a powerful platform for evaluating potential tumor markers and therapeutic targets in other to correlate cancer research. The database also serves as a tool for meta-analysis of the prognostic value of genes [92]. This database has the ability to evaluate the relationship between gene expression and patient prognosis across a large collection of cancer microarray datasets. The correlation between CHEK1 expression and survival was investigated in some selected solid tumors. The significance threshold was adjusted to a Cox *p*-value < 0.05.

### 4.4. Survival Analysis of CHEK1 in Some Selected Solid Tumors

The Kaplan–Meier plotter is a web-based tool accessed at http://kmplot.com/analysis/index.php?pbackground. The aim of this tool is meta-analysis-based discovery and validation of survival biomarkers. In this study, the correlations between CHEK1 expression and patient survival in gastric, breast, lung, and ovarian cancers were analyzed using the Kaplan–Meier plotter. According to various quantiles of biomarker expression, the tool divides patient samples into pairs of groups to analyze the prognostic value of a particular gene. The hazard ratio (HR) with 95% confidence intervals (CI) and log-rank *p*-value was also computed.

### 4.5. Complex Genomic Exploration of CHEK1

cBioPortal is an open-access resource for exploring multidimensional genomics data [93]. The database substantially reduces the drawback between complex genomic data and cancer researchers to provide high-quality access to molecular profiles and their clinical attributes. This further provides easy result interpretation of rich datasets into biologic insights and clinical application [93]. An integrative analysis of CHEK1 and clinical characteristics was performed using cBioPortal for Cancer Genomics at http://www.cbioportal.org/index.do, which is a web-based tool for the interactive visualization and analysis of multidimensional cancer genomics data sets [93,94]. The query interface combined with personalized data storage enables interactive investigations of genetic alterations of the CHEK1 gene across available samples. The primary search parameters included alterations (amplifications, deep deletions, and missense mutations), copy number alterations (CNAs) from GISTIC, and RNA sequencing data, using the default settings. For the secondary search, RNA sequencing data were considered.

### 4.6. Molecular Binding Analysis

The modified method of our previous analysis [56] was employed to investigate the mechanism of action of CHEK1-miR-195 and human argonaute protein in solid tumors. Schrodinger suit, MiRTarBase, Discovery Studio Visualizer (v19.1.0.18287), PROCHECK, PATCHDOCK, RNAfold, and RNA-composer software were all used in this analysis.

### 4.7. Statistical Analysis

All statistical analyses (Bar and forest plot) were performed using GraphPad Prism version 6 (GraphPad Software, La Jolla, CA, USA). Survival curves were plotted using the PrognoScan, cBioPortal, and Kaplan–Meier plotters. All results are displayed with *p*-values obtained from a long-rank test. The RMSD was set at 0.030 Å for the protein preparation. The protein quality check at PROCHECK was also considered significant at 85% and above for residues in the most favored regions. In PatchDock, RMSD was adjusted to 1.0 Å. Amino acid residues in binding were considered within the distance of 2–3.5 Å, while hydrogen bonding was considered between 2.0 Å. A *p*-value of < 0.05 were considered to be statistically significant.

## 5. Conclusions

Considering the development of cancer through multiple genes’ alteration, an increase in the copy number of a gene is an effective way to enhance cancer formation. The discovery of cancer-related genes could provide novel and successful cancer management. CHEK1 is an important irreplaceable gene. Together with the fatal effects of its deficiency in mammals, it could be used to explain the paucity of cancer-associated alterations of CHEK1. In this study, the CHEK1 gene was overexpressed in four different cancers versus normal tissues. Molecular docking based on microRNA gene regulation may ultimately lead to therapeutic strategies that target CHEK1 in solid tumors. This study suggests that CHEK1 could be further studied as a promising biomarker for cancer management.

## Figures and Tables

**Table 1:** Expression of CHEK1 in solid tumors (rank of 1%).

Cancer	Subtype	*p*-Value	Fold Change	Sample	Ref.
**Brain**	Glioblastoma	1.36 × 10^−26^	4.157	180	[27]
	Glioblastoma	4.67 × 10^−12^	−4.170	101	[28]
**Cervical**	Cervical Squamous Cell Carcinoma	7.71 × 10^−12^	2.319	45	[29]
**Colorectal**	Colon Adenoma	2.23 × 10^−12^	2.948	64	[30]
**Gastric**	Gastric cancer	2.23 × 10^−9^	2.409	180	[31]
	Gastric Intestinal Type Adenocarcinoma	2.99 × 10^−12^	3.936	69	[32]

**Table 2:** The relationship between CHEK1 expression and survival in solid tumor clinical samples.

Cancer	Dataset	Endpoint	PROBE ID	N	Cox *p*-value	HR
Bladder	GSE13507	Overall Survival	ILMN_1664630	165	0.040924	1.35
	GSE13507	Disease Specific Survival	ILMN_1664630	165	0.00706943	1.75
Brain	GSE4271-GPL96	Overall Survival	205394_at	77	0.000296208	1.92
Breast	GSE12276	Relapse Free Survival	205393_s_at	204	0.000166722	1.46
	GSE9195	Distant Metastasis Free Survival	205393_s_at	77	0.00319767	3.57
	GSE1456-GPL96	Relapse Free Survival	205394_at	159	0.00259906	2.11
	GSE1456-GPL96	Overall Survival	205393_s_at	159	0.0298328	1.78
	GSE1456-GPL96	Disease Specific Survival	205394_at	159	0.00442993	2.23
	GSE1456-GPL96	Overall Survival	205394_at	159	0.00383569	2.02
	GSE1456-GPL96	Relapse Free Survival	205393_s_at	159	0.000924772	2.44
	GSE1456-GPL96	Disease Specific Survival	205393_s_at	159	0.00145812	2.60
	GSE4922-GPL97	Disease Free Survival	238075_at	249	0.0101338	1.77
Colorectal	GSE14333	Disease Free Survival	205394_at	226	0.0121161	0.56
Lung	GSE13213	Overall Survival	A_23_P116123	117	0.00199884	1.40
	GSE31210	Overall Survival	238075_at	204	0.001305	2.39
	GSE31210	Relapse Free Survival	205393_s_at	204	0.000142	1.91
	GSE31210	Overall Survival	205394_at	204	0.000872076	1.93
Ovarian	DUKE-OC	Overall Survival	205394_at	133	0.0130251	0.71
	GSE8841	Overall Survival	2515	81	0.017998	1.91

**Table 3:** Genetic alteration summary of genes of interest in 12 solid tumors.

Cancer	CHEK1	ATM	CDC25A	CDC25C	EXO1	CLSPN	RAD51	ATR	TOPBP1	MCM4
**Bladder**	2%	14%	2.2%	1%	2%	4%	2.5%	9%	4%	7%
**Colon**	4%	13%	7%	4%	7%	10%	5%	6%	6%	1%
**Breast**	0.4%	2.9%	0.2%	0.4%	5%	0.6%	0.8%	1.2%	1.5%	5%
**Brain**	2.6%	1.4%	1.6%	0.2%	0.6%	0,4%	0.2%	0.8%	0.4%	0.4%
**Breast**	0.4%	0.5%	0.2%	0.6%	23%	0.7%	0.7%	5%	0.9%	12%
**Cervical**	4%	6%	1.4%	0.4%	2.2%	3%	1.4%	10%	9%	1.4%
**Stomach**	2.3%	12%	2.5%	2.1%	6%	3%	0.7%	8%	4%	6%
**Liver**	1.4%	5%	0.3%	1.4%	8%	0.6%	0.8%	4%	1.1%	7%
**Lung**	3%	10%	0.8%	2.2%	7%	2.4%	2.6%	4%	2.6%	6%
**Ovarian**	7%	11%	7%	4%	9%	12%	5%	21%	22%	8%
**Prostate**	16%	19%	22%	21%	28%	10%	9%	28%	27%	38%
**Pancreas**	9%	10%	4%	6%	11%	7%	6%	11%	8%	12%

The genetic alterations of all the genes of interest were further queried in the prostate cancer sample as shown in Figure 8. The Oncoprint feature of the cBioPortal was to determine the copy number alteration frequency of each individual gene within selected cancer subtypes.

**Table 4:** Docking scores (PatchDock).

Ago Complex	Score	Area	ACE
**miRNA-AGO**	20558	3146.10	−386.43
**miRNA-mRNA-AGO**	21006	3504.30	−275.84

AGO, argonaute; ACE, atomic contact energy.

**Table 5:** Amino acid residues of the binding pocket of the receptor protein involved in the molecular interaction with miR-195-5p and miR-195-5p-CHEK1 duplex (2.0 Å).

AGO Complex	Hydrophobic AA	Aromatic AA
**miR-195-5p**	(41) Ala221, Ile353, Ile365, Ile756, Val797	Tyr804, Phe811, Tyr815
**miR-195-5p-CHEK1**	(43) Val70, Val154, Ile159, Val347	Phe156

AA; Amino acid.

**Table 6:** Hydrogen bond interaction observed in the binding pocket of the human argonaute protein and miR-195-5p, andmiR-195-5p-CHEK1 within the distance of 2.0 Å.

Ago Complex	Atoms	AA Residues	Distance	NA Residues
**MiR-195-5p**	ARG351 LYS709 ARG761 ARG761 ASP358 THR361 ARG635 ASP436 HIS753	HH21—OP1 HZ2—OP2 H—OP1 HE—OP2 O—HO2′ HB—O2′ HD2—O3′ OD2—H5′2 ND1—H4′	2.0 1.4 1.9 1.3 1.3 1.5 1.5 1.8 1.9	C37 C34 C34 U35 G3 G3 U38 A12 G33
**miRNA-CHEK1**	ARG72 ARG69 LYS278 HIS56 ARG635 GLU157	HH12—OP1 HH21—O2′ O—H42 HE1—OP2 HD2—OP1 OE2—H5′	2.0 1.7 2.0 1.9 1.7 1.9	G20 G3 C4 U17 C9 A16

AA; Amino acid, NA; Nucleic acid.
